# Spectrum of pontocerebellar hypoplasia in 13 girls and boys with *CASK *mutations: confirmation of a recognizable phenotype and first description of a male mosaic patient

**DOI:** 10.1186/1750-1172-7-18

**Published:** 2012-03-27

**Authors:** Lydie Burglen, Sandra Chantot-Bastaraud, Catherine Garel, Mathieu Milh, Renaud Touraine, Ginevra Zanni, Florence Petit, Alexandra Afenjar, Cyril Goizet, Sabina Barresi, Aurélie Coussement, Christine Ioos, Leila Lazaro, Sylvie Joriot, Isabelle Desguerre, Didier Lacombe, Vincent des Portes, Enrico Bertini, Jean-Pierre Siffroi, Thierry Billette de Villemeur, Diana Rodriguez

**Affiliations:** 1 Centre de Référence Maladies Rares « malformations et maladies congénitales du cervelet », Hôpital Trousseau-Paris, CHU de Lyon, CHU de Lille, Paris, France; 2 AP-HP, Hôpital Trousseau, Service de Génétique et d'Embryologie Médicales, Paris, France; 3 Inserm U676, Hôpital Robert Debré, Paris, France; 4 AP-HP, Service de Radiologie, Hôpital Trousseau, Paris, France; 5 AP-HM, Hôpital La Timone Enfants, Service de Neuropédiatrie, Marseille, France; 6 Service de Génétique Médicale, Centre Hospitalier Universitaire, St Etienne, France; 7 Unit of Molecular Medicine for Neuromuscular and Neurodegenerative Disorders, Department of Neurosciences, Bambino Gesù Pediatric Research Hospital, Rome, Italy; 8 Service de Génétique Médicale, Centre Hospitalier Universitaire, Lille, France; 9 AP-HP, Service de Neuropédiatrie, Hôpital Trousseau, Paris, France; 10 CHU Bordeaux, Service de Génétique Médicale, Université Bordeaux, Laboratoire Maladies Rares: Génétique et Métabolisme (MRGM), Bordeaux EA4576, France; 11 AP-HP, Service de cytogénétique, Hôpital Cochin, Paris, France; 12 AP-HP, Pôle de Pédiatrie, Hôpital Raymond Poincaré, Garches, France; 13 Service de pédiatrie, Centre hospitalier, Bayonne 64109, France; 14 Service de Neuropédiatrie, Centre Hospitalier Universitaire, Lille, France; 15 AP-HP, Service de Neuropédiatrie, Hôpital Necker, Paris, France; 16 Service de Neuropédiatrie, CHU de Lyon, et Université Lyon1, Lyon, France; 17 UPMC Univ Paris 06, Paris, France

**Keywords:** Pontocerebellar hypoplasia, Microcephaly, *CASK *gene, Mosaicism, Array-CGH

## Abstract

**Background:**

Pontocerebellar hypoplasia (PCH) is a heterogeneous group of diseases characterized by lack of development and/or early neurodegeneration of cerebellum and brainstem. According to clinical features, seven subtypes of PCH have been described, PCH type 2 related to *TSEN54 *mutations being the most frequent. PCH is most often autosomal recessive though *de novo *anomalies in the X-linked gene *CASK *have recently been identified in patients, mostly females, presenting with intellectual disability, microcephaly and PCH (MICPCH).

**Methods:**

Fourteen patients (12 females and two males; aged 16 months-14 years) presenting with PCH at neuroimaging and with clinical characteristics unsuggestive of PCH1 or PCH2 were included. The *CASK *gene screening was performed using Array-CGH and sequencing. Clinical and neuroradiological features were collected.

**Results:**

We observed a high frequency of patients with a *CASK *mutation (13/14). Ten patients (8 girls and 2 boys) had intragenic mutations and three female patients had a Xp11.4 submicroscopic deletion including the *CASK *gene. All were *de novo *mutations. Phenotype was variable in severity but highly similar among the 11 girls and was characterized by psychomotor retardation, severe intellectual disability, progressive microcephaly, dystonia, mild dysmorphism, and scoliosis. Other signs were frequently associated, such as growth retardation, ophthalmologic anomalies (glaucoma, megalocornea and optic atrophy), deafness and epilepsy. As expected in an X-linked disease manifesting mainly in females, the boy hemizygous for a splice mutation had a very severe phenotype with nearly no development and refractory epilepsy. We described a mild phenotype in a boy with a mosaic truncating mutation. We found some degree of correlation between severity of the vermis hypoplasia and clinical phenotype.

**Conclusion:**

This study describes a new series of PCH female patients with *CASK *inactivating mutations and confirms that these patients have a recognizable although variable phenotype consisting of a specific form of pontocerebellar hypoplasia. In addition, we report the second male patient to present with a severe MICPCH phenotype and a *de novo CASK *mutation and describe for the first time a mildly affected male patient harboring a mosaic mutation. In our reference centre, *CASK *related PCH is the second most frequent cause of PCH. The identification of a *de novo *mutation in these patients enables accurate and reassuring genetic counselling.

## Background

Pontocerebellar hypoplasia (PCH) is a heterogeneous group of diseases characterized by lack of development and/or early neurodegeneration of cerebellum and brainstem. Indeed, PCH has been associated with chromosomal abnormalities or metabolic disorders as well as congenital muscular dystrophies [1-5]. According to clinical features, seven subtypes of PCH have been described (Table 1) [6-21].

**Table 1:** Classification of PCH

PCH	Type 1	Type 2	Type 3	Type 4	Type 5	Type 6	Type 7
Distinctive features	Anterior horn cell degeneration	Dyskinetic movements Seizures (frequent)	Seizures Short stature Optic atrophy	Severe prenatal form of PCH2 with: Polyhydramnios Contractures Myoclonus Apneic episodes Early postnatal death	Severe prenatal form of type 2 with: Fetal onset of seizure-like activity Early postnatal death	Severe neonatal encephalopathy with: Hypotonia, and inconstantly Intractable seizures Edema Increased lactate Mitochondrial respiratory chain defects	Hypotonia Apneic episodes Seizures Vanishing testis

Inheritance	AR	AR	AR	AR	AR	AR	Unknown

Genes or loci	*VRK1*	*TSEN54*	7q11-21	*TSEN54*	*TSEN54*	*RARS2*	unknown
	*TSEN54*	*TSEN34*					
	*RARS2*	*TSEN2*					

References	[6-11]	[6,7,11,12]	[11,13,14]	[7,11,12,15]	[11,16,17]	[11,18-20]	[11,21]

In addition to the distinctive features reported in the table, microcephaly and intellectual disability are present in all types of PCH. AR: autosomal recessive

Recently, the identification of several novel responsible genes has added new insight to the characterization of this group of disorders: three out of four subunits of the tRNA splicing endonuclease complex, *TSEN54*, *TSEN34 *and *TSEN2 *have been shown to be involved in PCH2, PCH4, PCH5 and in one patient with PCH1 [10,12,17]. PCH6 as well as some PCH1 are related to mutations in the mitochondrial arginyl-tRNA synthetase *RARS2 *gene [18-20]. The *VRK1 *gene was identified as responsible for PCH1 but the reported patients seemed to be affected by a very mild condition compared to the clinical description of classical PCH1 [10]. In addition, similar neuroimaging aspects of the cerebellum and brainstem have been observed in patients with large deletions/mutations of the *VLDLR *gene and in *Reelin*-associated lissencephaly with PCH [22-25].

More recently *de novo *anomalies in the X-linked gene *CASK *(calcium/calmodulin-dependent serine protein kinase) have been identified in patients with PCH, mostly females, displaying intellectual disability and microcephaly (MICPCH syndrome) [26-29].

In this study, we performed a systematic screening for *CASK *mutations and deletions in 14 patients presenting with PCH at neuroimaging but for whom the clinical features differed from PCH1 or PCH2 (PCH4 and PCH5). We observed a high frequency of *CASK *abnormalities (13/14). We describe the clinical aspects of these patients, including the second reported male patient with an extremely severe PCH and clinical phenotype and the first mosaic mutation found in a mildly affected male. Finally, we discuss the variability of the clinical and radiological phenotypes and highlight the characteristics of this recognizable syndrome.

## Methods

### Patients

We studied fourteen patients (12 females and two males) referred to our reference centre for cerebellar malformations. One girl was included in this series following the Array-CGH identification of a Xp11.4 submicroscopic deletion including *CASK *in the course of investigations for her intellectual disability. All other patients (13) were investigated for the genetic diagnosis of their PCH. Each patient had been examined by at least one of us. Neither anterior horn cell disease nor choreic movements were observed, ruling out PCH type 1 or 2. Before this study, the entire coding region of the gene *TSEN54*, responsible for PCH2, had been systematically sequenced in five of the patients and was normal. MRI scans were systematically reviewed in all patients. We have previously reported briefly two of the female patients (Patients 4 and 10) [27]. For each patient and their parents, blood samples were collected after obtaining written informed consent. The investigations fulfilled our institutions' ethical rules for human studies.

### Molecular analysis

Mutation analysis was systematically performed in all patients, except in patient 1 in whom the Xp11.4 deletion was previously found. The 27 *CASK *coding exons and flanking intronic regions were PCR-amplified and sequenced using 27 primer pairs (sequences available on request). When a mutation was identified in a patient, parental DNAs were analyzed. Parental identities were checked using 16 highly polymorphic markers (PowerPlex 16 System, Promega). X-inactivation was determined by methylation analysis of the androgen receptor polymorphic CAG repeat and the *FMR1 *gene CGG repeats.

Whole blood sample (2.5 ml) from patient 8 was collected into a PAXgene Blood RNA tube (PreAnalytiX; Qiagen). Total RNA was isolated using the PAXgene Blood RNA Kit (Qiagen) following manufacturer's guidelines and subjected to reverse transcription with the SuperScript II Reverse Transcriptase kit (Invitrogen). Specific primers for PCR amplification of *CASK *mRNA were designed in exon 23 and exon 25, respectively, based on their sequence data from the GenBank database (GenBank accession number NM_003688.3). Analysis of PCR products was performed by purifying single bands from agarose gel using a gel extraction kit (QiaQuick gel extraction kit; Qiagen), and sequenced directly.

### Array-CGH analysis

Four patients were found to be negative after mutation screening and, therefore, were analyzed by Array-CGH (Additional file 1).

## Results

Overall, a *CASK *anomaly was identified in 13/14 patients (10 intragenic mutations and three Xp11.4 submicroscopic deletions).

### *CASK *molecular analysis

**Figure 1:**
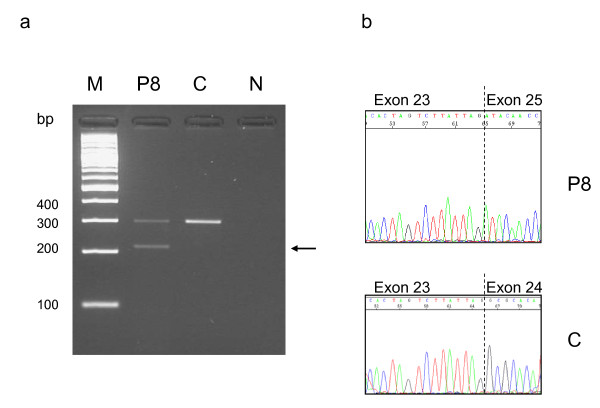
**RT-PCR analysis in patient 8 (mutation c.2302 + 5 G > A), showing exon skipping and aberrant transcript**. a. RT-PCR products were amplified from cDNA of patient 8 and from one healthy individual using primers in exon 23 and in exon 25. The *arrow *indicates an aberrant sized product in patient 8 in addition to normal sized transcript observed in patient 8 and in healthy control. *M *marker, *P8 *patient 8, *C *control, *N *negative control. **b**. The PCR products were purified and sequenced. Sequence chromatograms from normal sized amplicon with normal sequence exon 23-24-25, and from abnormal amplicon, showing absence of exon 24.

**Figure 2:**
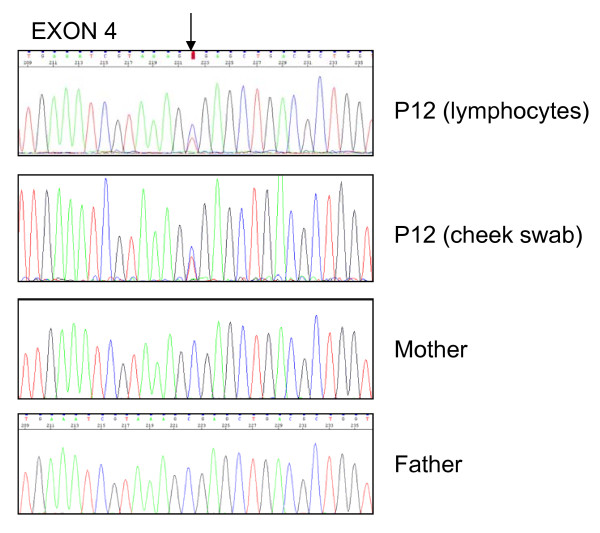
**Sequence analysis of exon 4 amplified from DNA obtained from lymphocytes and cheek swab of patient 12 and from lymphocytes of his parents**. The upper two sequence profiles (patient) show low signals for the mutant variant superimposed on the wild-type sequence (arrow). The lower two sequence profiles (parents) show the wild type sequence.

Ten *CASK *mutations, including eight novel mutations, were identified in 10 patients (eight females and two males). Several types of mutations were observed: five nonsense, one frameshift and four splice-site mutations (Table 2). In all cases, parental DNA analysis confirmed paternity and the *de novo *occurrence of the mutation. Three of the four intronic mutations (patients 5, 9 and 13) were localized at the consensus donor or acceptor canonical splice site and prediction software programs predicted that the respective donor or acceptor site was broken (Additional file 2). mRNA from these patients was not available to perform CASK transcript analysis. The splice mutation in patient 8 involved the fifth nucleotide of intron 24 and we used four different splice-site prediction software programs to predict the effect of this mutation. The results obtained from these *in silico *tools were consistent since they all predicted that the wild type donor site was broken (Additional file 2). RT-PCR performed on mRNA from lymphocytes of this patient revealed an aberrant transcript with skipping of exon 24 (Figure 1). This aberrant transcript is in-frame and is predicted to produce a protein which lacks 28 amino acids with the insertion of a Asp residue. The exact pathogenic mechanism of this mutation is unknown. In patient 12 a heterozygous profile was observed suggesting a mosaic nonsense mutation in this male patient. This profile was confirmed by analysis of a second tissue (cheek swab) (Figure 2). Finally, apart from the splice mutation in patient 8, all mutations were classified as truncating mutations. *CASK *sequencing in patients 2, 3 and 14 DNA was normal.

**Table 2:** Clinical and molecular data of our 13 patients with *CASK *mutation

Patient	1	2	3	4	5	6	7	8	9	10	11	12	13
Sex/age at last examination (years)	F/7	F/3	F/14	F/13	F/3	F/1	F/1	F/10	F/14	F/8	F/3.5	M/15	M/1.3

Birth (weeks)	39	40	40	40	40	39	41	40	41.5	40	39	41	40

Pregnancy	pre-eclampsia	N	N	IUGR	INT	N	N	N	microcephaly IUGR	N	N	N	N

OFC/weight/length at birth (SD)	-2/-1/-1	-3/-1/-1	-1/-1/-1	-1/-1/-1	-2/-1/-1.5	-1.5/-1/-1	-2/0/-0.5	-1/0/-0.5	-3/-2/-3	-1/-1/-1.5	0/0/0	-3/-1.5/-2	-2/0/-0.5

feeding difficulties	++	+	+	+	NA	+	-	-	+	+	+	+	++

Severe developmental delay/intellectual disability	+	+	+	+	+	+	+	+	+	+	+	+	+

Language	bable	bable	bable	bable	NA	TY	TY	2 words	5-6 words	10 words	-	12 words	-

Ability to hold his head	+	+	+	+	+	+	+	+	+	+	NA	+	-

Ability to sit (age in months)	13	-	36	20	18	-	-	24	18	18	27	18	-

Ability to stand (age in years)	3	-	5 (briefly)	4 (briefly)	NA	TY	TY	-	-	2	NA	1.5	-

Method of movement	walks on 4 legs wheelchair	rolling	wheelchair	wheelchair	walks with support wheelchair	TY	TY	wheelchair	wheelchair	walks, runs	wheelchair	walks	bedridden

Hand control	+	-	+	+	+	+	NA	+	+	+	NA	+	-

Spasticity	++	+	+	++	+	NA	+	++	++	-	NA	-	+++

Dystonia	-	+	+	+	+	NA	+	+	+	-	NA	+	+++

Choreoathethosis	-	-	-	-	-	-	-	-	-	-	NA	-	-

Epilepsy	-	-	-	+	-	-	+	+	-	-	-	-	+++

Stereotypies	-	NA	+	+	NA	NA	-	-	+	+	+	+	-

OFC/height (SD)	-6/-3	-6/-3.5	-6/-2	-7,5/-4	-5/NA	-3/1	-4/-1	-6.5/-4.5	-6/-4	-4/-3	-5/0	-3.5/-3.5	-6/-2

ophthalmologic anomalies	strabismus	ret	OA	meg, OA SP ret low vision	ret	meg bil glau	-	myopia	SP ret low vision	-	strabismus	-	OA

Abnormal ERG	-	+	NA	+	+	NA	NA	-	++	+	NA	NA	-

Hearing	N	unil SN deaf	NA	N	unil SN deaf	NA	N	bil SN deaf	N	N	N	N	-

Sleep disturbance	++	++	++	+	NA	NA	++	-	++	++	++	-	-

Scoliosis	+	+	+	+	NA	NA	-	-	+	-	-	-	-

Extremities	N	N	N	long fingers and toes	N	long fingers	N	N	clinodactyly, overlapping toes	hypoplastic nails	slender hands and feet	2-3 syndactyly	long slender fingers with retractions

Dysmorphism	NA	+	+	+	NA	+	+	+	+	+	NA	+	+

Mutation	Xp11.4 deletion 0.3 Mb	Xp11.3-p11.4 deletion 3 Mb	Xp11.4 deletion 0.5 Mb	c.1968 G > A (p.Trp656*)	c.2040-2A > G	c.2080 C > T (p.Gln694*)	c.2074 C > T (p.Gln692*)	c.2302 + 5 G > A	c.2039 + 1 G > T	c.1970 G > A (p.Trp657*)	c.1501dupA (p.Met501fs)	c.[ =/316 C > T] (p.Arg106*) mosaic	c.278 + 1 G > A

Exon/intron	exons 1-8	exons 1-27	exon 1	exon 21	intron 21	exon 22	exon 22	intron 24	intron 21	exon 21	exon 15	exon 4	intron 3

X-inactivation	NA	random	random	random	random	random	random	random	random	random	NA	na	na

Parents	*de novo*	NA	*de novo*	*de novo*	*de novo*	*de novo*	*de novo*	*de novo*	*de novo*	*de novo*	*de novo*	*de novo*	*de novo*

The mutation nomenclature follows the mutation nomenclature rules of the Human Genome Variation Society http://www.hgvs.org  version 2.0 and is based on RefSeq NM_003688.3 with +1 as the A of the ATG initiation codon

F: female; M: male; N: Normal; IUGR: intrauterine growth retardation; INT: increased nuchal translucency; +: present. ++: present, moderate; +++: present, severe; -: absent; NA: non available; TY: too young; ret: retinopathy; OA: optic atrophy; meg: megalocornea; SP ret: salt-and-pepper retinopathy; glau: glaucoma; bil: bilateral; unil: unilateral; SN deaf: sensorineural deafness; na: non applicable

### Array results

Array-CGH was performed in four patients. Since the phenotype of patients 2 and 3 was strikingly similar to that of patients with *CASK *mutations, we performed Array-CGH in order to detect a submicroscopic deletion involving *CASK*. In patient 1 Array-CGH was performed as part of the diagnostic workshop of her intellectual disability with pontocerebellar hypoplasia. An Xp11.4 deletion involving part of or the entire *CASK *gene was detected in these three girls. The largest, a 2.9 Mb Xp11.4-p11.3 deletion, included *CASK *as well as eight RefSeq adjacent genes: *USP9X, DDX3X, NYX, GPR34, GPR82, MAOA, MAOB*, and *NDP *(patient 2). The 575 kb deletion in patient 3 included the exon 1 of *CASK *only but no others RefSeq genes. The smallest deletion (patient 1) was 359 kb in size and removed the eight first exons of *CASK *as well as the RefSeq genes *GPR34 *and *GPR82*. FISH analysis confirmed the deletions in patients 1 and 3 whilst revealing a normal result for their parents. Finally Array-CGH was performed in patient 14 in spite of a different, more severe, phenotype and was normal. Array-CGH results are summarized in Additional file 1.

### X-inactivation study

X-inactivation study was performed on 9/11 female patients with *CASK *anomalies and showed random X-inactivation.

### Clinical phenotype of the 13 patients with *CASK *anomalies

Clinical features are summarized in Table 2.

### Phenotype of the 11 female patients

**Figure 3:**
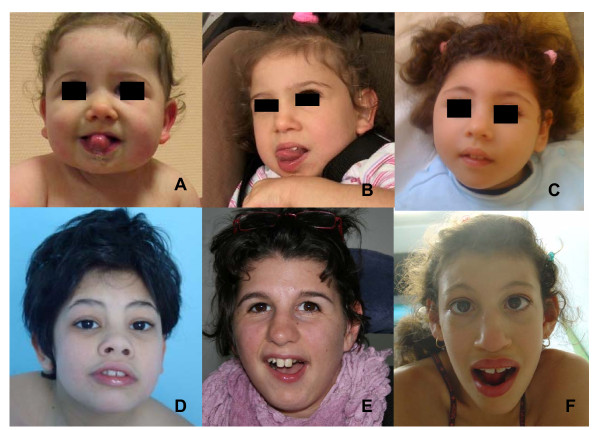
**Facial features of *CASK*-positive patients**. A and B: patient 8 (1 year (A) and 4 years (B)). C: patient 7 (18 months). D: patient 12 (13 years). E: patient 9 (13 years). F: patient 4 (12 years). Note minor facial dysmorphism: round face, small chin, well-drawn eyebrows in the younger patients; longer face, high and large nasal bridge, long nose, protuding maxilla, in the older patients. Signed informed consent was obtained from the parents of the affected children for publication of the images.

Birth occipitofrontal head circumference (OFC) was in the normal or low normal range in all patients. Feeding difficulties were frequently (8/11) noticed during the first months of life (gastroesophageal reflux, sucking and swallowing difficulties). In four patients these difficulties and excessive drooling persisted at last examination (3-14 years) and one patient had been fed via a gastrostomy tube since the age of five years. Progressive microcephaly was present in all patients. Psychomotor development was variable but intellectual development was severely impaired in all patients. All patients had eye contact and acquired head control in the age range of three to 24 months. Purposeful hand control was achieved in six patients. Among the nine patients older than three years, eight were able to sit (acquired at one to three years), only one was able to walk unaided and seven patients were wheelchair bound. Language was nearly absent in 7/9 of the older patients and reduced to a few words in two. Neurologic examination displayed extrapyramidal symptoms (dystonia, buccolingual dyskinesia) and/or spasticity in 8/11. One patient had axial hypotonia and peripheral hypertonia in the first years but presented with near normal neurological examination at 11 years. The behavioral phenotype included sleep disturbances (multiple awakening during the first years) in at least seven patients and hand stereotypies and self-biting in five. Epilepsy was present in three patients: two had atypical absences and patient 7 had West syndrome in infancy. One patient presented with disabling massive myoclonias. Scoliosis and constipation were frequent. Various ophthalmologic anomalies were noted: retinopathy (5, all had abnormal ERG and two had salt-and-pepper retinopathy), optic atrophy (2), glaucoma/megalocornea (2) and nystagmus (1). Three patients had sensorineural hearing loss. One patient developed type 1 diabetes mellitus at the age of 14 years. Minor dysmorphism was observed: round face tending to lengthen in older patients, well-drawn arched eyebrows, large nasal bridge, protruding maxilla and maxillar incisors in older patients (Figure 3). No other visceral malformation was detected in any patient.

### Phenotype of the two male patients

Patient 13 presented with severe neonatal hypotonia, along with intractable seizures, distal contractures, long fingers and diaphragmatic eventration. OFC was initially normal, followed by postnatal microcephaly, reaching -6SD at the age of 16 months. Minor dysmorphic features were noted: retrognathism, high arched palate, downslanting palpebral fissures, broad nasal bridge, low-set ears with prominent lobules, hypoplastic scrotum, edema of the hands and feet. He had no visual contact and communication skills were limited to smiling. Examination showed profound hypotonia with no head control, spastic tetraparesia and severe dystonia with opisthotonos. Seizures were refractory to treatment and consisted of daily spasms and tonic seizures with suppression-burst. He had severe gastroesophageal reflux and swallowing difficulties requiring gastrostomy. Ophthalmologic examination showed very hypoplastic and pale optic discs. Visual evoked responses were absent. Brainstem auditory evoked potential showed normal wave I but nearly absent central responses.

Patient 12 was born at term after a normal pregnancy with normal parameters. His psychomotor development was delayed and walking was achieved at four years. At age 15, he had severe intellectual disability, his language was limited to a few words but he used non verbal communication tools. He was able to walk independently, had dystonia, stereotypic movements and buccofacial dyskinesia. His OFC and height reached -3.5 SD. Ophthalmological examination and auditory testing were normal.

### MRI features

**Figure 4:**
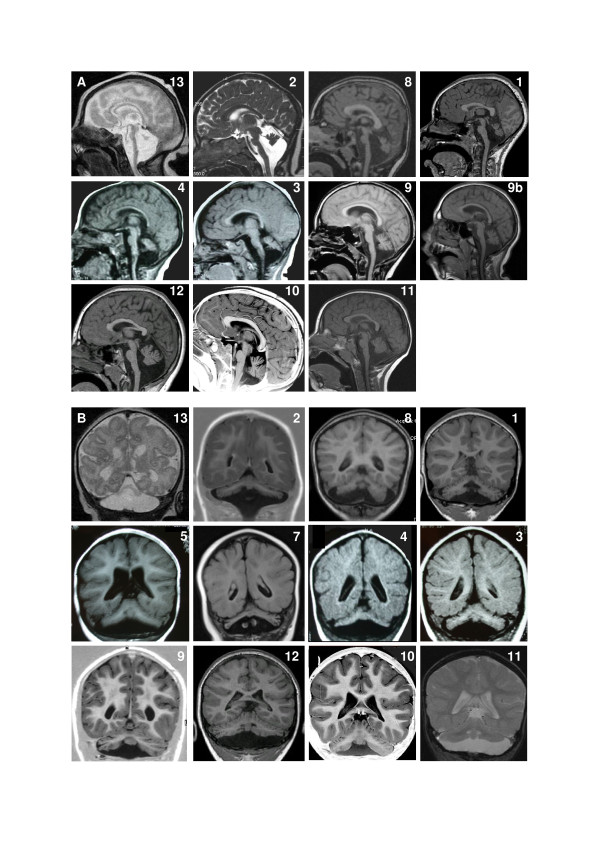
**MRIs in patients**. **A**. Sagittal images showing spectrum of vermis and pons hypoplasia. Number represents the number of the patient. Figure 9 shows MRI of patient 9 at age 4 months and figure 9b patient 9 at age 11 years. Note that in all patients, the pons is very small but has a relative sparing of his buldging, mainly in its superior part. Hypoplasia predominates at the inferior part of the pons. Vermis hypoplasia is very variable, severe in patient 13, very slight in patient 10-11-12 and predominates at the inferior part. V4 is open in most cases. **B**. Coronal images showing spectrum of cerebellar hemispheric hypoplasia. Number represents the number of the patient. Hemispheres are frequently asymmetric. Note that the vermis does not protrude from the hemispheres indicating similar involvement of the vermis and the hemispheres. This pattern is different from that of PCH2 in which the vermis is relatively spared leading to the classic image of "dragonfly", the protruding vermis being the body of the dragonfly and the hemispheres, the wings. There is no progression of the lesions between successive MRI in patient 9.

In all patients, MRI showed hypoplasia of the brainstem and cerebellum (Figure 4). The brainstem hypoplasia was associated with sparing of the superior part of the pons and mainly concerned the inferior part. The cerebellar hypoplasia involved similarly the vermis and hemispheres which were often asymmetric. The severity of cerebellar hypoplasia was extremely variable, allowing the definition of a severity gradient. Patient 13 had the most severe cerebellar involvement with near absent cerebellar hemispheres. His brainstem was very thin but with preservation of slight anterior relief at the upper part of the pons. At the opposite end of the spectrum, patients 9 to 12 had mild cerebellar hypoplasia. In three patients anomalies were stable on two successive MRIs. Gyration and basal ganglia nuclei were normal.

## Discussion

Calcium/calmodulin-dependent serine protein kinase (CASK) is a member of the membrane-associated guanylate kinase (MAGUK) family involved in synapse formation and in the regulation of gene expression, including Reelin which is critical in brain development [30].

Using Array-CGH, two groups identified Xp11.4 submicroscopic deletions involving the *CASK *gene in girls affected with intellectual disability and microcephaly [31,32]. Najm et al. reported three novel female patients with *CASK *deletions or disruption and described more precisely the phenotype including cerebellar and brainstem hypoplasia [26]. Furthermore in two patients without Xp11.4 deletions they identified *de novo CASK *intragenic mutations. One of the patients was a boy who was affected with a very severe form of PCH. Thirty-three further female patients with PCH and *CASK de novo *loss-of-function anomalies have been reported [27-29]. In addition, *CASK *hypomorphic inherited mutations have been described in patients, mainly male, affected with intellectual disability and not featuring MICPCH [33-35].

In this study, we describe 13 patients (11 girls and 2 boys) affected with PCH and harboring heterozygous or hemizygous *CASK *anomalies. Among a large series of PCH patients that were referred to us, we prioritized the screening of the *CASK *gene in these patients since clinical criteria for PHC1 and PCH2 were absent. We particularly noted the differences in the clinical features between our patients and those of PCH2 patients. Indeed, patients had no incessant choreic movements, were hypotonic rather than hypertonic, had a less severe psychomotor retardation and had features not usually observed in PCH2 (sensorial involvement as well as slight dysmorphism). Sequencing allowed us to identify 10 *CASK *intragenic mutations, in eight girls and two boys. All mutations were private, with no identified hotspot. Nine were inactivating mutations and one was predicted to lead to a large in-frame deletion. In three other female patients Array-CGH analysis revealed a Xp11.4 submicroscopic deletion involving part of or the entire *CASK *gene.

The patients with a *CASK *mutation showed a characteristic phenotype with some variability concerning the severity and the associated sensorial involvement. All had neonatal feeding difficulties, intellectual and motor disabilities and progressive microcephaly. Most had profound disabilities but two patients are able to use a few words and have acquired autonomous walking. Postnatal microcephaly was severe in seven patients (OFC < -6SD) but OFC ranged between -3 to -4 SD in four. Four patients had epilepsy and this was refractory in one. Ophthalmological abnormalities were present in at least six patients and sensorineural hearing loss in two. The association with a diabetes mellitus observed in one patient has not been previously reported and could be co-incidental. All our patients with a *CASK *mutation presented slight but homogeneous dysmorphic features. As reported by Moog et al., we observed a broad nasal bridge in the majority of patients and a micrognathism in the younger. A round face tending to lengthen in older patients, well-drawn arched eyebrows and protruding maxilla and maxillar incisors in older patients represented the major facial findings in our series. These findings were also frequently observed in patients presented by Najm et al and Moog et al [26,29].

In one patient we failed to detect any *CASK *mutation or deletion. This patient had a very severe phenotype characterized by severe encephalopathy, almost no psychomotor acquisition, epilepsy, absence of choreoathetosic movements and premature death. MRI revealed severe pontocerebellar hypoplasia as well as severe subtentorial atrophy. A previous pregnancy has been terminated because of PCH suggesting the possibility of recessive disease.

All our patients had severe intellectual and motor disabilities with PCH but at varying degrees. A correlation between the clinical severity and the MRI findings may be discussed. This correlation is obvious for the two male patients, notably due to the severity of patient 13. He had the most severe clinical features including refractory epilepsy, optic atrophy and almost no psychomotor acquisition at age of 16 months and his MRI showed the most severe PCH. By contrast, the male with a mosaic mutation (patient 12) was able to walk from the age of three years and use a few words. He had no epilepsy or spasticity, his microcephaly was moderate (-3.5 SD) and his MRI showed mild cerebellar hypoplasia. This correlation is less clear however in female patients. Nevertheless, the female patient with the mildest clinical features, patient 10, had a clinical phenotype very similar to that of the male patient 12 and also had mild cerebellar hypoplasia. Moreover, in previously reported female patient series the acquisition of walking and the ability to use a few words seems to correlate with a less severe cerebellar hypoplasia (Patient 6 in Hayashi at al.; patients 7, 15, 20, 21 and 24 in Moog et al) [28,29]. However this clinicoradiologic correlation cannot be relied upon entirely since patient 11, while still young, had a mild cerebellar hypoplasia but she was unable to walk. As reported by Takanashi et al., we observed a reduced area of the cerebrum, pons and cerebellum, but a sparing of the corpus callosum in most patients [36].

Aside from this partial clinicoradiologic correlation, we failed to identify any clear genotype-phenotype correlation in this series, except for the non mosaic male patient, who is hemizygous for the splice mutation and has the most severe phenotype. The severity of the disease in patients with a *CASK *deletion was very similar to patients with intragenic mutations regarding motor and intellectual disability, epilepsy, MRI anomalies and hearing and visual defects, even when the deletion involved nine other genes. Furthermore, two very close mutations (p.Trp657* and p.Trp656*) were observed in two unrelated girls: one had the milder phenotype, acquired independent walking at age 3, was able to run and to use a few words at 8 years and the second was a severely affected girl who had acquired no language and was unable to walk.

Finally, all girls and boys with inactivating *CASK *mutations presented with a recognizable phenotype characterized by severe postnatal microcephaly, moderate to severe motor and intellectual disability, feeding and sleep difficulties, frequent ophthalmological anomalies and PCH on MRI. The absence of anterior horn involvement excluded PCH1. Severe post-natal microcephaly and dystonia as well as some neuroradiological features (dragonfly-like cerebellum, hypoplasia of the pons) could be suggestive of PCH2. However, PCH2 and *CASK*-related PCH are distinct entities. Indeed, PCH2 patients have a more severe clinical course (near absent development and choreic movements), and do not present with ophthalmologic abnormalities (glaucoma, megalocornea, optic atrophy, and retinopathy), hearing loss or facial dysmorphism, which are frequently found in *CASK *mutated patients. Furthermore, analysis of the MRI gives additional clues showing relative sparing of the pons, asymmetry of the cerebellar hemispheres as well as vermian and cerebellar involvement. In PCH2 there is a relative sparing of the vermis but cerebellar hemispheres and brainstem are severely affected with a flat pons [9,29,37]. If there is any doubt, the major *TSEN54 *mutation (A307S) observed in a majority of PCH2 patients, can easily be ruled out.

Only three male patients with MICPCH and *de novo CASK *mutations have been reported [this report, 26] suggesting a possible lethality in most affected male patients. The two male patients with MICPCH and non mosaic *CASK *mutations presented with a severe PCH, severe neurological impairment with near absent development, absence of sucking and swallowing, optic atrophy, refractory epilepsy and edema of the hands and feet in one [26]. This phenotype is reminiscent of PEHO syndrome but the presence of severe PCH unlike progressive cerebellar atrophy should prompt to look for *CASK *mutations [38].

To date, two groups have reported molecular analysis results in their cohort of PCH patients [9,27]. These studies showed that the majority (45 to 63%) of patients were affected with PCH2/4 with mutations in one *TSEN *gene, mostly the A307S mutation in *TSEN54*. In our cohort of 40 PCH patients referred to our reference center, *CASK*-related PCH was the second most frequent cause (13/40, 32%) behind PCH2/4 (18/40, 45%, manuscript under preparation).

Several groups have reported *CASK *mutations in male patients with other phenotypes: FG syndrome and X-linked mental retardation (XLMR) with or without nystagmus [33-35]. The mutations reported in these patients were missense or splicing mutations resulting in in-frame deletions and were all familial. The affected boys had mild to severe intellectual disability without severe motor impairment and without microcephaly. Nystagmus was a frequent feature and some patients had seizures, strabismus, optic disc pallor, visual impairment or dysmorphic features. In those families, most females had no (10/13) or mild (2/13) intellectual disability and one was severely retarded. Data concerning MRI are available in only 2/25 male patients, one of whom has been described as normal and the other showing cerebellar hypoplasia and pachygyria [35]. The male patient with cerebellar hypoplasia had severe intellectual disabilities, nystagmus and a small OFC at the 3^rd ^centile, but no motor abnormalities or seizures were reported and his mutation was inherited. More details concerning the MRI features of male patients with hypomorphic *CASK *mutations would be contributive to determine which mentally retarded patients should be tested for *CASK *mutations.

Therefore the spectrum of phenotypes associated with *CASK *mutations is very large in males, from lethality or severe neurologic impairment with PCH to isolated intellectual disability. In females, depending on the type of mutation, it extends from normal to severely mentally retarded patients with motor disabilities related to PCH.

## Conclusion

Genotype-phenotype correlations in our patients and previously published patients confirm that *CASK *inactivating mutations are *de novo *and are associated with PCH and microcephaly in females and a severe phenotype in males while missense mutations or in-frame deletions are observed in males and rarely in females with variable degrees of XLMR with or without nystagmus.

As patients with non-inactivating mutations have a non specific intellectual disability, it seems difficult to determine the clinical criteria which would allow the selection of patients for sequencing, particularly in sporadic cases. On the other hand, patients with inactivating mutations have a recognizable phenotype consisting of a specific form of PCH. Consequently *CASK *analysis should be performed in girls presenting with intellectual disability, microcephaly and PCH, in the absence of symptoms suggesting PCH2 (severe encephalopathy with choreoathetosis) and particularly in the presence of ophthalmological anomalies such as optic atrophy or glaucoma. Furthermore we consider that *CASK *exonic deletion or duplication screening must be part of the strategy when looking at *CASK *anomalies in a patient with a suggestive phenotype. The identification of a *de novo *mutation allows to give a reassuring genetic counseling, whilst PCH is most often autosomal recessive.

## Abbreviation

PCH: Pontocerebellar hypoplasia.

## Competing interests

DL is on scientific advisory board of Genzyme. The other authors declare that they have no competing interests.

## Authors' contributions

LB designed and coordinated the study, saw some of the patients, revised all the clinical and MRI data, carried out the molecular genetic studies and wrote the manuscript. SCB and AC carried out Array-CGH analysis and co-wrote the manuscript. CGa revised all MRIs and co-wrote the manuscript. RT performed X-inactivation study, collected the clinical data of one patient and revised the manuscript. MM, FP, AA, CGo, SB, CI, LL, SJ, ID, DL, VDP contributed to the clinical work, seeing patients and collecting clinical data. GZ and EB contributed to the acquisition and analysis of the clinical and molecular data in one patient, and co-wrote the manuscript. JPS and TBV contributed towards analyzing the data and revising the manuscript. DR designed and co-coordinated the study, contributed to the acquisition and analysis of the clinical and MRI data and co-wrote the manuscript. All authors read and approved the final manuscript.

**Table 3:** *CASK *intragenic mutations identified in our series and in literature (ref seq NM_003688.3)

Type of mutation	Exon	Mutation	Protein change predicted	Family	Sex	Phenotype	Reference
Nonsense	2	c.79 C > T	p.Arg27*	**nd**	F	MICPCH	[28]

	4	c.316 C > T	p.Arg106*	*de novo/de novo*	F/M	MICPCH	[28], This study

	5	c.379 C > T	p.Glu127*	nd	F	MICPCH	[29]

	17	c.1639 C > T	p.Gln547*	nd	F	MICPCH	[29]

	21	c.1915 C > T	p.Arg639*	*de novo*	F	MICPCH	[26]

	**21**	**c.1968 G > A**	p.Trp656*	***de novo***	**F**	MICPCH	[27]**, This study**

	**21**	**c.1970 G > A**	p.Trp657*	***de novo***	**F**	MICPCH	[27], **This study**

	22	c.2074 C > T	p.Gln692*	nd/*de novo*	F	MICPCH	[29], This study

	**22**	**c.2080 C > T**	p.Gln694*	***de novo***	**F**	MICPCH	**This study**

	**27**	c.2632 C > T	p.Gln878*	*de novo*	F	MICPCH	[28]

Frameshift	**2**	c.68delT	p.Phe23fs	*de novo*	F	MICPCH	[29]

	**3**	c.243_244delTA	p.Tyr81*	nd	F	MICPCH	[28]

	**15**	**c.1501dupA**	**p.Met501fs**	***de novo***	**F**	MICPCH	**This study**

	16	c.1578delG	p.Arg526fs	*de novo*	F	MICPCH	[27]

Splice defect	I2	c.173-2A > C	*Skipping of exon 3 leading to premature stop codon*	*de novo*	F	MICPCH	[29]

	**I3**	**c.278 + 1 G > A**	*Exon skipping ?^a^*	***de novo***	**M**	MICPCH	**This study**

	I4	c.357-1 G > A	*Skipping of exon 5 or skipping of exon 5* *and insertion of partial intron 5*	*de novo*	F	MICPCH	[28]

	I5	c.430-2A > T	*Exon skipping ?^a^*	nd	F	MICPCH	[29]

	I8	c.831 + 2 T > G	*Exon skipping ?^a^*	*de novo*	F	MICPCH	[29]

	9	c.915 G > A	*Skipping of exon 9 leading to an in-frame deletion* *of 28 amino-acids*	Mother normal	M	MICPCH	[26]

	I17	c.1668 + 1 G > A	*Exon skipping ?^a^*	*de novo*	F	MICPCH	[29]

	**I21**	**c.2039 + 1 G > T**	*Exon skipping ?^a^*	**de novo**	F	MICPCH	**This study**

	**I21**	c.2040-1 G > C	*Skipping of partial or entire exon 22*	de novo	F	MICPCH	[28]

	**I21**	**c.2040-2A > G**	*Exon skipping ?^a^*	**de novo**	F	MICPCH	**This study**

	22	c.2129A > G	p.710_718del *splicing defect leading to an in-frame deletion of 9 amino acids*	familial	M	ID-nystagmus	[33,35]

	**I24**	**c.2302 + 5 G > A**	*Skipping of exon 24 leading to an in-frame deletion of 28 amino acids and insertion of a Asp residue*	**de novo**	F	MICPCH	**This study**

	I25	c.2521-2A > T	*Splicing defect leading to 2 in-frame deletions (3 amino acids; 28 amino acids)*	familial	M	ID-nystagmus	[35]

Missense	2	c.83 G > T	p.Arg28Leu	Familial	M/F	FG syndrome	[34]

	8	c.802 T > C	p.Tyr268His	Familial	M	ID-nystagmus	[33,35]

	13	c.1186 C > T	p.Pro396Ser	Familial	M/F	ID	[33,35]

	23	c.2168A > G*	p.Tyr723Cys^b^	Familial	M/F	ID-nystagmus	[35]

	27	c.2740 T > C**	p.Trp914Arg^c^	Familial	M	ID	[33,35]

In bold, mutations found in our patients (Mutations are named according to the guidelines of the Human Genome Variation Society (HGVS) Version 2.0)

^a^Experimental data non available. ^b^This mutation was reported as Y728C by Hackett according to reference sequence ENST00000378163 but the nucleotide substitution is c.2168A > G and the mutation Y723C according to the reference sequence NM_003688.3. ^c^This mutation was reported as W919R by Hackett according to reference sequence ENST00000378163 but the nucleotide substitution is c.2740 T > C and the mutation W914R according to the reference sequence NM_003688.3. nd: not done. F: female; M male; MIC-PCH: mental retardation, microcephaly and pontocerebellar hypoplasia; I: intron; ID: intellectual disability

## Supplementary Material

Additional file 1 **Results of Array-CGH**. All deletions were mapped according to the March 2006 assembly of the UCSC genome browser (NCBI Build 36.1/hg18; http://genome.ucsc.edu). Click here for file

Additional file 2 **Prediction of pathogenicity obtained for the four intronic mutations using four splice-site prediction software programs**. Click here for file
