# Supplementary material for: Spectrum of pontocerebellar hypoplasia in 13 girls and boys with CASK mutations: confirmation of a recognizable phenotype and first description of a male mosaic patient
Source: Orphanet J Rare Dis. 2012 Mar 27;7:18. doi: 10.1186/1750-1172-7-18 (PMC3351739; doi:10.1186/1750-1172-7-18)
Supplement: Additional file 1 — Results of Array-CGH. All deletions were mapped according to the March 2006 assembly of the UCSC genome browser (NCBI Build 36.1/hg18; http://genome.ucsc.edu). [file 1750-1172-7-18-S1.DOC]

| patient | platform | deletion | Size (Mb) | Start | End | BAC clone for FISH analysis | Status of inheritance |
| --- | --- | --- | --- | --- | --- | --- | --- |
| 1 | Puce Nimblegen Whole genome tiling v2.0 | Xp11.4 | 0.359 | 41,403,101 | 41,762,580 | RP3-421H1 | *de novo* |
| 2 | Agilent Human Genome CGH Microarray Kit 244K* | Xp11.4-p11.3 | 2.9 | 40,930,084 | 43,870,425 | NA | NA |
| 3 | HumanCytoSNP-12 BeadChip (http://www.illumina.com) | Xp11.4 | 0.575 | 41,627,459 | 42,202,811 | RP11-394N9 | *de novo* |
| 14 | HumanCytoSNP-12 BeadChip (http://www.illumina.com) | No deletion |  |  |  |  |  |

*Data analysis was performed using the Mapix Software v 4.6.2 and Nexus Copy Number software v4 (Biodiscovery Inc, EI Segundo, California, USA).

NA: Peripheral blood lymphocytes for FISH experiment and parental DNA were not available for validation experiments in patient 2.
