# Supplementary material for: Spectrum of pontocerebellar hypoplasia in 13 girls and boys with CASK mutations: confirmation of a recognizable phenotype and first description of a male mosaic patient
Source: Orphanet J Rare Dis. 2012 Mar 27;7:18. doi: 10.1186/1750-1172-7-18 (PMC3351739; doi:10.1186/1750-1172-7-18)
Supplement: Additional file 2 — Prediction of pathogenicity obtained for the four intronic mutations using four splice-site prediction software programs. [file 1750-1172-7-18-S2.DOC]

|  | Patient 5  c.2040-2A>G | Patient 8  c.2302+5G>A | Patient 9  c.2039+1G>T | Patient 13  c.278+1G>A |
| --- | --- | --- | --- | --- |
| NNsplice 09 - Berkely Drosophila Genome Project (BDGP) ([www.fruitfly.org/seq_tools/splice.html](http://www.fruitfly.org/seq_tools/splice.html)); | Acceptor site broken | Donor site broken | Donor site broken | Donor site broken |
| NetGene 2 (NG2)  ([www.cbs.dtu.dk/services/NetGene2/](http://www.cbs.dtu.dk/services/NetGene2/)) | Acceptor site broken | Donor site broken | Donor site broken | Donor site broken |
| MaxEntScan (MES) (<http://genes.mit.edu/burgelab/maxent/Xmaxentscan_scoreseq.html>) | Acceptor site broken  Score 8.47 to -3.20 | Donor site broken  Score 6.58 to -3.67 | Donor site broken  Score 8.35 to -0.15 | Donor site broken Score 7.54 to -0.83 |
| Human Splicing Finder version 2.4  ([www.umd.be/HSF/](http://www.umd.be/HSF/)) | Acceptor site broken  Score 77.09 to 48.14  Variation -37.55 | Donor site broken  Score 74.29 to 45.34  Variation -38.97 | Donor site broken  Score 85.49 to 58.66  Variation -31.39 | Donor site broken  Score 82.27 to 55.43  Variation -32.62 |
